# The global, regional, and national burden of colorectal cancer and its attributable risk factors in 204 countries and territories, 1990-2021: a systematic analysis for the global burden of disease study 2021

**DOI:** 10.3389/fonc.2025.1665430

**Published:** 2025-11-19

**Authors:** Shen Tian, Yun-Shuai Wang, Dong Wei

**Affiliations:** 1 Department of Central Laboratory, Luoyang Central Hospital Affiliated to Zhengzhou University, Luoyang, Henan, China; 2 Department of General Surgery, Luoyang Central Hospital Affiliated to Zhengzhou University, Luoyang, Henan, China

**Keywords:** GBD, colorectal cancer, SDI, risk factor, DALYs

## Abstract

**Background:**

Colorectal cancer (CRC) is among the leading causes of cancer-related mortality worldwide. This study aimed to assess the global burden of CRC across 204 countries and territories from 1990 to 2021, and identify its attributable risk factors.

**Methods:**

Estimates of CRC incidence, mortality, and disability-adjusted life years (DALYs) were derived from the Global Burden of Diseases, Injuries, and Risk Factors Study (GBD) 2021, stratified by age, sex, and geographical region over the 1990–2021 period. Additionally, DALYs attributable to risk factors with established causal links to CRC were calculated.

**Results:**

100,000 population (95% uncertainty interval [UI]: 25.36–29.90), mortality rate 13.23 per 100,000 (95% UI: 12.04–14.19), and DALY rate 309.21 per 100,000 (95% UI: 287.52–331.52). The number of new CRC cases increased from 916,583 in 1990 to 2,194,143 in 2021. The age-standardized incidence rate (ASIR) rose from 24.0 to 25.6 per 100,000. High SDI (Socio-demographic Index) regions had the highest ASIR (40.5 per 100,000 in 2021), while low SDI regions had the lowest (7.4 per 100,000 in 2021). Between 1990 and 2021, the global age-standardized mortality rate (ASDR) decreased from 15.6 to 12.4 per 100,000, and the age-standardized DALY rate declined from 357.3 to 283.2 per 100,000. However, this reduction was uneven across SDI regions. Key risk factors included behavioral and metabolic factors, among which a diet low in whole grains significantly increased CRC incidence.

**Conclusion:**

From 1990 to 2021, the global CRC burden increased significantly, with notable variations across SDI regions. While high SDI regions made progress in reducing mortality and DALYs, low SDI regions now face a heavier burden. Targeted interventions for modifiable risk factors and improved healthcare access in less developed regions are essential to mitigate the global impact of CRC.

## Introduction

Colorectal cancer (CRC) is a leading cause of cancer mortality worldwide. Together with other gastrointestinal malignancies (gastric cancer, esophageal cancer, pancreatic cancer, hepatocellular carcinoma), it accounts for approximately one-third of all cancer-related DALYs (1–4). As a common digestive tract malignancy, CRC ranks third in global incidence and second in mortality among all malignant tumors (5, 6). In 2018, the International Agency for Research on Cancer reported that the highest incidence rates of colon cancer were observed in Europe, North America, Australia, New Zealand, and East Asia, and in 2020, CRC emerged as the world’s third most prevalent cancer, with 2 million new cases recorded that year, it was also among the top causes of cancer-related deaths (7, 8).

The etiology and pathogenesis of CRC remain incompletely understood, but can be broadly categorized into genetic factors, environmental factors, and lifestyle conditions (9, 10). CRC incidence has risen annually, paralleling the growing prevalence of high-fat, low-fiber diets. While tobacco and alcohol are established risk factors for several cancers, the mechanism linking smoking to CRC remains unclear (11–13).

Using data from the Global Burden of Diseases, Injuries, and Risk Factors Study (GBD) 2021, this analysis presents estimates of CRC incidence, mortality, ASIR, ASDR, and DALY rates across 204 countries and territories from 1990 to 2021, stratified by age and sex. It also reports age-standardized DALYs by SDI and estimates the proportion of DALYs attributable to key CRC risk factors (tobacco smoking, alcohol consumption, low physical activity, high body mass index [BMI]). The goal is to provide actionable insights for policymakers, funders, and researchers to develop region-specific interventions, optimize healthcare resource allocation, and guide future CRC research.

## Methods

### Study data

Annual CRC incident cases and age-standardized incidence rates (1990–2021), stratified by sex, region, and country, were extracted from GHDx (http://vizhub.healthdata.org/gbd-results). 204 different country/territories were included in the data, which were divided into five SDI strata(high, middle, middle, low, and middle)and twenty-one geographic regions(e.g., East Asia). Incidence data derived from individual or aggregated registries, including CI5, SEER, EUERG, and NORDCAN.

### Statistical analysis

We quantified trends in CRC incidence using the age-standardized incidence rate (ASIR) and estimated annual percentage change (EAPC) (14). Standardization is essential when comparing populations with divergent age structures or monitoring the same population over time as age distributions shift. The age-standardized rate (ASR, per 100,000 population) was calculated via the direct method: the sum of products of age-specific rates (*a_i_*, where *i* denotes the *i^th^* age class) and corresponding weights (*w_i_*) from the selected reference standard population, divided by the total weight of the reference population, i.e.,


$$\text{ASR}=\frac{\sum_{i=1}^{A}a_{i}w_{i}}{\sum_{i=1}^{A}a_{i}}\times 100,000$$

EAPC is a summary and widely used measure of the ASR trend over a specified interval. A regression line was fitted to the natural logarithm of the rates i.e., y=α+βx+ϵ, where y=ln(ASR), and x=calendar year (15). If the EAPC estimation and the lower boundary of its 95% CI were both > 0, the ASR was deemed to be in an increasing trend (16). In contrast the ASR was in a decreasing trend if the EAPC estimation and the upper boundary of its 95% CI were both < 0. Otherwise, the ASR was deemed to be stable over time.

Data cleaning, computations, and graph generation were performed using R software (version 4.4.1). Visualizations were created using the ggplot2 package and final changes were done using Adobe Illustrator 2020.

## Results

### Colorectal cancer incidence burden

**Figure 1:**
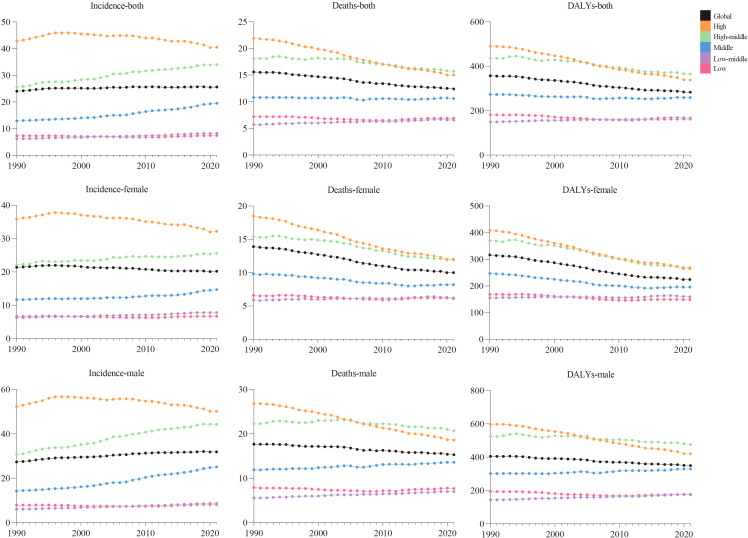
Trends in global incidence, deaths, and DALY rates of colorectal cancer by sex and SDI, from 1990 to 2021.

Globally, new CRC cases surged from 916.58×10^3^ (1990) to 2194.14×10^3^ (2021), with ASIR rising modestly from 24.0 to 25.6 per 100,000 (EAPC = 0.15, 95% CI:0.12–0.19), indicating stability (**Figure 1**, **Table 1**). Incidence increased across all five SDI regions: in 2021, high SDI had the most cases (839.75×10^3^, 95% UI:764.45×10^3^–885.16×10^3^) and highest ASIR (40.5 per 100,000, 95% UI:37.4–42.4); low SDI had the lowest ASIR (7.4 per 100,000, 95% UI:6.7–8.2). Middle SDI showed the largest relative rise (3.5-fold, EAPC = 1.38, 95% CI:1.3–1.46).

**Table 1:** Incidence of colorectal cancer in 1990 and 2021, and the EAPC from 1990 to 2021.

Characteristics	1990		2021		1990-2021
	Incidence cases	ASIR	Incidence cases	ASIR	EAPC
	No.×10^3^(95%UI)	(95%UI)	No.×10^3^(95%UI)	(95%UI)	(95%CI)
Global	916.58 (866.24-951.9)	24.0 (22.5-25)	2194.14 (2001.27-2359.39)	25.6 (23.3-27.5)	0.15 (0.12-0.19)
Socio-demographic index					
High	475.23 (449.62-489.57)	42.8 (40.5-44.1)	839.75 (764.45-885.16)	40.5 (37.4-42.4)	-0.25 (-0.35--0.16)
High-middle	251 (237.46-263.21)	25.6 (24.1-26.8)	669.66 (598.31-746.4)	34.0 (30.3-38)	0.93 (0.89-0.97)
Middle	134.43 (121.31-148.57)	12.9 (11.6-14.2)	526.19 (462.02-595.12)	19.5 (17.1-22)	1.38 (1.3-1.46)
Low-middle	38.29 (33.16-43.28)	6.2 (5.4-6.9)	119.42 (109.34-130.92)	8.2 (7.5-9)	0.96 (0.93-0.99)
Low	16.47 (12.93-18.87)	7.3 (5.8-8.4)	36.65 (32.71-40.88)	7.4 (6.7-8.2)	-0.06 (-0.18-0.06)
Sex					
Female	446.82 (414.14472.55-)	21.4 (19.7-22.6)	930.68 (824.67-1017.65)	20.2 (17.9-22)	-0.29 (-0.33--0.25)
Male	469.76 (445.31-492.3)	27.3 (25.9-28.5)	1263.46 (1146.5-1400.38)	31.9 (29-35.3)	0.5 (0.46-0.54)
Region					
High-income Asia Pacific	79.54 (75.5-82.39)	39.7 (37.4-41.2)	207.28 (179.5-223.33)	44.9 (40.2-47.8)	0.33 (0.23-0.43)
Central Asia	6.18 (5.77-6.59)	12.9 (12-13.8)	8.89 (7.94-9.81)	10.8 (9.7-11.9)	-0.15 (-0.35-0.05)
East Asia	165.08 (142.14-189.66)	19.1 (16.5-21.8)	684.93 (559.52-823.3)	31.6 (25.9-37.8)	1.75 (1.66-1.84)
South Asia	28.14 (24.02-31.93)	4.7 (4-5.3)	85.12 (6.61-95.25)	5.6 (5.1-6.3)	0.46 (0.33-0.59)
Southeast Asia	29.32 (24.9-33.18)	11.3 (9.6-12.7)	116.94 (101.26-132.26)	17.7 (15.4-19.9)	1.45 (1.4-1.5)
Australasia	11.84 (10.91-12.8)	50.6 (46.6-54.6)	23.28 (20.5-26.41)	44.0 (38.9-49.6)	-0.58 (-0.71--0.45)
Caribbean	6.21 (5.82-6.61)	24.1 (22.6-25.7)	18.48 (16.07-20.94)	34.3 (29.9-38.9)	1.26 (1.19-1.34)
Central Europe	42.18 (40.23-43.86)	28.3 (27-29.5)	85.87 (79.29-92.79)	38.8 (35.7-42)	0.98 (0.82-1.14)
Eastern Europe	71.75 (69.02-73.94)	25.5 (24.5-26.3)	113.25 (104.41-122.49)	32.1 (29.6-34.7)	0.62 (0.51-0.73)
Western Europe	243.93 (229.6-253.38)	41.8 (39.5-43.4)	375.46 (337.71-401.85)	40.5 (37.2-43.1)	-0.11 (-0.26-0.04)
Andean Latin America	1.9 (1.65-2.19)	9.5 (8.2-10.9)	8.45 (6.7-10.53)	14.4 (11.4-17.9)	1.43 (1.33-1.53)
Central Latin America	7.63 (7.31-7.9)	9.3 (8.9-9.7)	44.55 (39.67-49.72)	17.7 (15.8-19.8)	2.05 (1.99-2.11)
Southern Latin America	10.98 (10.13-11.89)	24.1 (22.1-26.1)	24.75 (21.92-27.56)	28.3 (25.1-31.5)	0.73 (0.56-0.9)
Tropical Latin America	9.84 (9.35-10.32)	11 (10.3-11.6)	44.24 (40.86-47.14)	17.2 (15.8-18.3)	1.42 (1.31-1.54)
North Africa and Middle East	17.57 (14.96-19.79)	10.4 (9.1-11.6)	66.09 (58.13-74.94)	14.4 (12.7-16.3)	1.31 (1.16-1.47)
High-income North America	167.65 (155.82-174.74)	47.3 (44.2-49.3)	244.68 (226.55-256.38)	38.8 (36.1-40.5)	-0.8 (-0.92--0.67)
Oceania	0.2 (0.16-0.24)	6.8 (5.7-8)	0.48 (0.41-0.56)	6.4 (5.5-7.4)	-0.18 (-0.27--0.1)
Central Sub-Saharan Africa	1.59 (1.27-1.96)	7.4 (6.1-9)	4.21 (3.21-5.57)	7.9 (6.1-10.5)	0.24 (0.06-0.41)
Eastern Sub-Saharan Africa	8.18 (6.32-9.31)	11.1 (8.7-12.5)	17.95 (15.71-20.78)	11.2 (9.8-12.8)	-0.1 (-0.21-0.01)
Southern Sub-Saharan Africa	2.52 (2.23-3.06)	9.5 (8.4-11.6)	7.62 (6.88-8.47)	13.4 (12.2-14.8)	1.29 (1.07-1.51)
Western Sub-Saharan Africa	4.35 (3.71-5.1)	5.2 (4.4-6)	11.62 (9.67-13.67)	6.3 (5.4-7.3)	0.78 (0.71-0.85)

*ASR, age-standardised rate; EAPC, estimated annual percentage change; CI, confidence interval; UI, uncertainty interval.

Among 21 GBD regions, incidence rose universally, with peak EAPC in Central Latin America (2.05, 95% CI:1.99–2.11) and trough in high-income North America (−0.8, 95% CI:−0.92 to −0.67). In 2021, high-income Asia Pacific (44.9 per 100,000, 95% UI:40.2–47.8) and Western Europe (44.0 per 100,000, 95% UI:38.9–49.6) had highest ASIR; low-income regions (Oceania:6.4; Western Sub-Saharan Africa:6.3; South Asia:5.6 per 100,000) the lowest.

**Figure 2:**
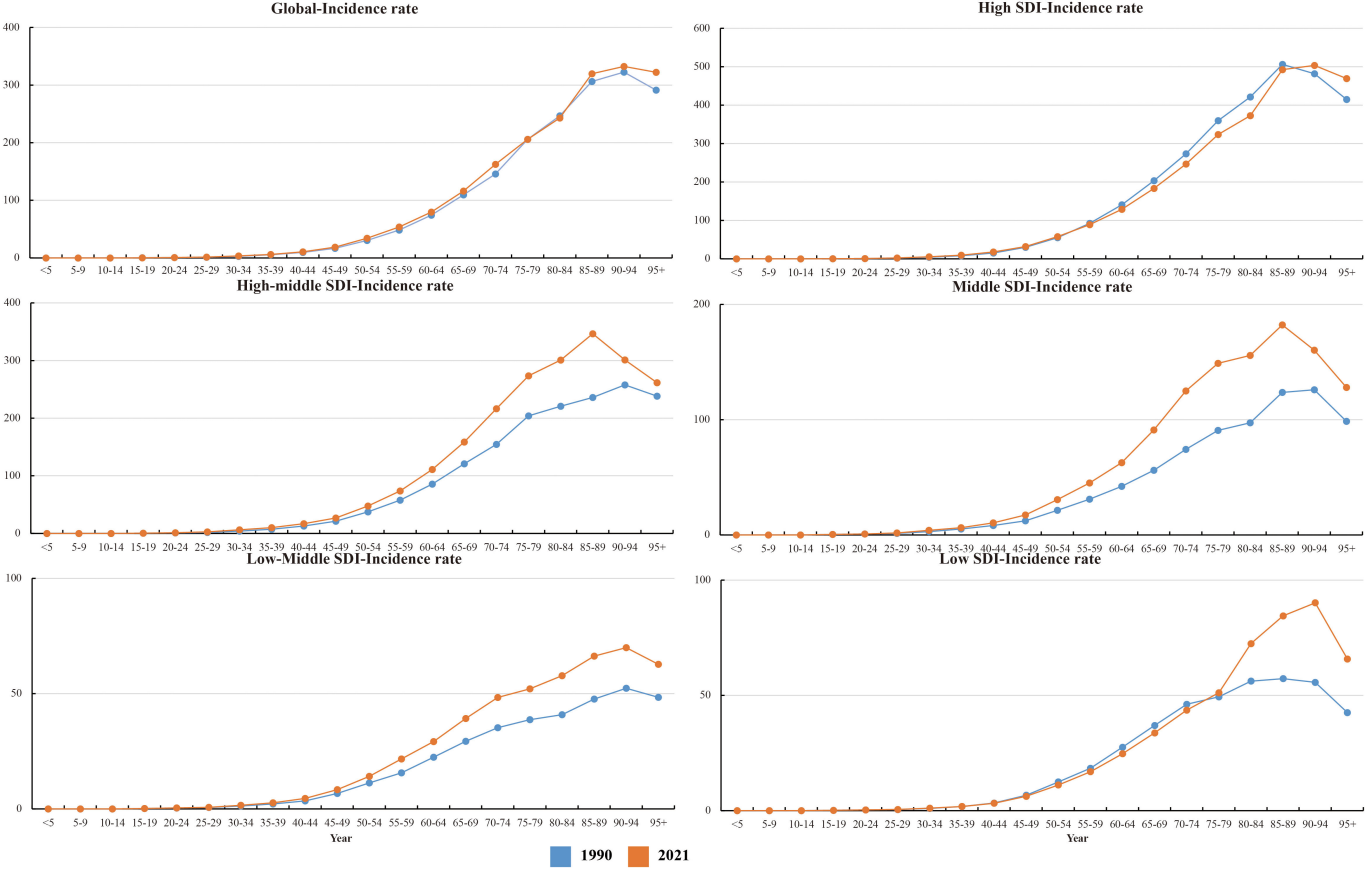
Age-standardized rates (per 100,000 population) of colorectal cancer among regions based on SDI in 2021. **(a)** ASIR. **(b)** ASDR. **(c)** Age-standardized DALY rate.

**Figure 3:**
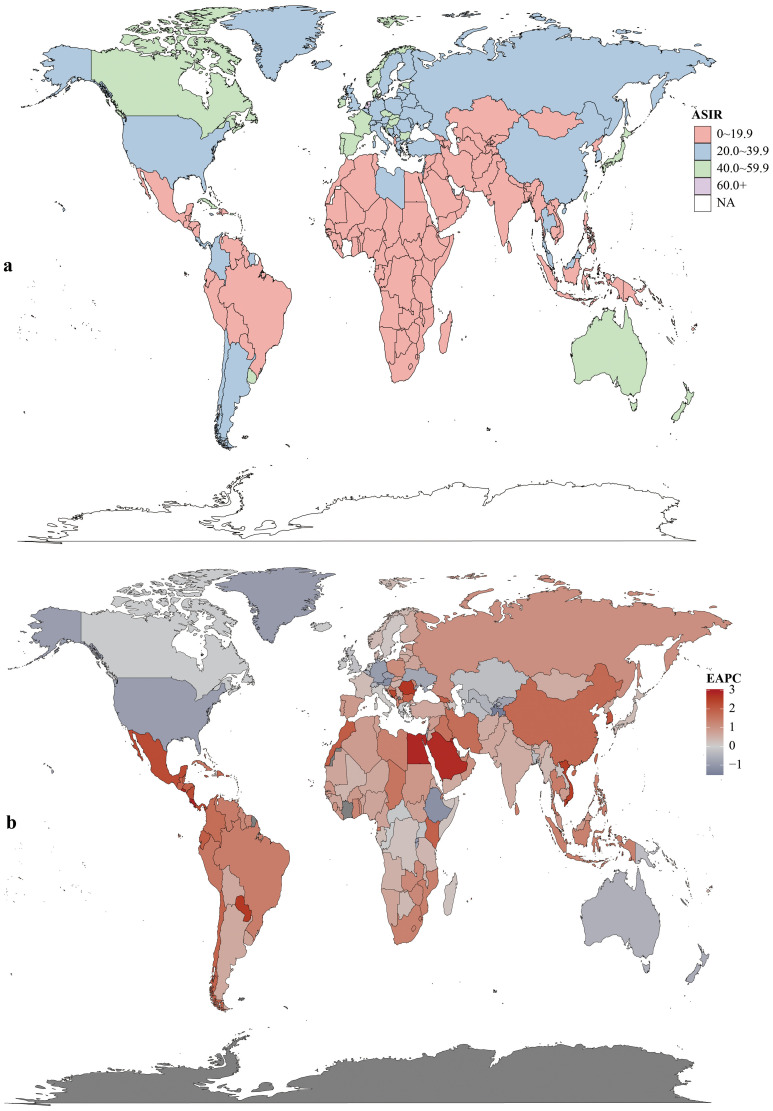
Incidence rates **(a)** and estimated annual percentage change (EAPC) **(b)** of colorectal cancer in 204 countries and territories.

Males bore higher burden. ASIR/ASDR correlated positively with SDI (**Figure 2**), with sex disparities (**Supplementary Figures S5**, **S6**). In 2021, the US, China, Japan had most new cases; Tokelau, Niue, Nauru the fewest. The Netherlands, Monaco, Bermuda had highest ASIRs; The Gambia, Papua New Guinea, Mozambique the lowest (**Figure 3**, **Supplementary Table S1**).

### Colorectal cancer deaths and DALY burden

In 2021, global CRC deaths totaled 1044.07×10^3^ (95% UI:950.19×10^3^–1120.17×10^3^), with ASDR at 12.4 per 100,000 (95% UI:11.2–13.3) – a 3.2 per 100,000 reduction from 1990 (**Figure 1**, **Table 2**). Global 2021 CRC DALYs: crude rate 244.01 (95% UI:226.89–261.62) and age-standardized rate 283.2 per 100,000 (95% UI:263.1–303.3), down 74.1 per 100,000 since 1990 (**Figure 1**, **Table 3**).

**Table 2:** Deaths of colorectal cancer in 1990 and 2021, and the EAPC from 1990 to 2021.

Characteristics	1990		2021		1990-2021
	Deaths cases	ASDR	Deaths cases	ASDR	EAPC
	No.×10^3^(95%UI)	(95%UI)	No.×10^3^(95%UI)	(95%UI)	(95%CI)
Global	570.32 (536.54-597.67)	15.6 (14.5-16.3)	1044.07 (950.19-1120.17)	12.4 (11.2-13.3)	-0.81 (-0.84--0.77)
Socio-demographic index					
High	243.22 (227.49-251.65)	21.9 (20.4-22.6)	336.57 (299.02-359.61)	15.0 (13.6-15.9)	-1.31 (-1.34--1.27)
High-middle	171.22 (160.93-179.67)	18.1 (17-19)	308.17 (278.04-338.27)	15.7 (14.1-17.3)	-0.52 (-0.59--0.46)
Middle	105.72 (95.51-116.51)	10.8 (9.8-11.8)	274.87 (243.37-306.69)	10.6 (9.4-11.8)	-0.1 (-0.13--0.06)
Low-middle	33.89 (29.28-38.36)	5.7 (5-6.4)	91.27 (83.73-99.85)	6.6 (6-7.2)	0.48 (0.45-0.51)
Low	15.46 (12.11-17.69)	7.2 (5.7-8.2)	31.85 (28.5-35.51)	6.9 (6.2-7.6)	-0.22 (-0.33--0.12)
Sex					
Female	282.61 (258.89-301.4)	13.9 (12.7-14.8)	462.51 (407.3-503.54)	10.0 (8.8-10.8)	-1.19 (-1.24--1.14)
Male	287.71 (269.82-304.98)	17.7 (16.7-18.7)	581.56 (528.25-641.42)	15.3 (13.9-16.9)	-0.5 (-0.52--0.47)
Region					
High-income Asia Pacific	35.64 (33.64-36.95)	18.5 (17.2-19.2)	80.69 (67.27-88.00)	15.0 (13-16.1)	-0.73 (-0.78--0.69)
Central Asia	4.83 (4.5-5.16)	10.4 (9.6-11.1)	6.15 (5.49-6.77)	7.9 (7.1-8.7)	-0.5 (-0.64--0.35)
East Asia	123.64 (106.93-141.86)	15.4 (13.4-17.6)	287.88 (235.56-343.28)	13.8 (11.3-16.3)	-0.44 (-0.5--0.39)
South Asia	25.28 (21.6-28.74)	4.4 (3.8-5.1)	66.94 (60.2-74.84)	4.6 (4.2-5.2)	0.02 (-0.08-0.13)
Southeast Asia	24.78 (20.97-28.07)	10.1 (8.7-11.4)	79.42 (68.45-89.29)	12.7 (11.1-14.3)	0.74 (0.67-0.8)
Australasia	5.78 (5.3-6.25)	24.9 (22.8-26.9)	8.28 (7.18-9.41)	14.6 (12.8-16.5)	-1.92 (-2.01--1.83)
Caribbean	3.47 (3.24-3.72)	14.0 (13-14.9)	7.88 (6.87-8.97)	14.6 (12.7-16.6)	0.27 (0.24-0.31)
Central Europe	32.12 (30.6-33.4)	22.0 (21-22.9)	51.84 (47.75-55.68)	22.6 (20.8-24.3)	-0.03 (-0.15-0.09)
Eastern Europe	50.1 (48.18-51.69)	18.1 (17.3-18.6)	64.37 (59.12-69.82)	18.1 (16.6-19.6)	-0.19 (-0.3--0.08)
Western Europe	136.38 (127.11-142.17)	23 (21.5-23.9)	156.64 (136.86-169.94)	15.1 (13.5-16.3)	-1.4 (-1.45--1.36)
Andean Latin America	1.72 (1.5-1.97)	8.9 (7.7-10.1)	5.78 (4.6-7.05)	10.0 (8-12.2)	0.46 (0.36-0.57)
Central Latin America	5.62 (5.37-5.82)	7.2 (6.9-7.5)	22.93 (20.34-25.52)	9.3 (8.3-10.4)	0.86 (0.78-0.94)
Southern Latin America	8.97 (8.27-9.71)	20.1 (18.5-21.8)	16.12 (14.31-18.00)	18.1 (16.1-20.2)	-0.1 (-0.26-0.07)
Tropical Latin America	8.1 (7.66-8.51)	9.6 (8.9-10.1)	29.41 (26.98-31.34)	11.6 (10.6-12.3)	0.66 (0.57-0.75)
North Africa and Middle East	14.11 (12.13-15.84)	9.0 (7.9-10.1)	37.39 (32.76-42.27)	8.9 (7.9-10.1)	0.21 (0.06-0.35)
High-income North America	73.96 (67.85-77.46)	20.6 (19-21.5)	85.87 (77.87-90.93)	13 (11.9-13.7)	-1.63 (-1.69--1.57)
Oceania	0.17 (0.14-0.2)	6.3 (5.4-7.4)	0.38 (0.32-0.45)	5.6 (4.8-6.4)	-0.37 (-0.47--0.27)
Central Sub-Saharan Africa	1.49 (1.2-1.84)	7.4 (6.1-9)	3.69 (2.8-4.93)	7.5 (5.8-10.1)	0.06 (-0.09-0.21)
Eastern Sub-Saharan Africa	7.78 (6-8.84)	11.1 (8.7-12.5)	15.97 (13.94-18.32)	10.8 (9.4-12.2)	-0.21 (-0.3--0.12)
Southern Sub-Saharan Africa	2.23 (1.97-2.72)	8.8 (7.8-10.9)	6.13 (5.55-6.79)	11.5 (10.4-12.6)	0.97 (0.71-1.24)
Western Sub-Saharan Africa	4.16 (3.56-4.87)	5.2 (4.5-6)	10.31 (8.67-12.1)	6.0 (5.1-6.9)	0.62 (0.55-0.68)

*ASR, age-standardised rate; EAPC, estimated annual percentage change; CI, confidence interval; UI, uncertainty interval.

**Table 3:** DALYs of colorectal cancer in 1990 and 2021, and the EAPC from 1990 to 2021.

Characteristics	1990		2021		1990-2021
	DALYs (per 100,000 persons)	ASR	DALYs (per 100,000 persons)	ASR	EAPC
	No.(95%UI)	(95%UI)	No.(95%UI)	(95%UI)	(95%CI)
Global	143.97 (135.69-151.67)	357.3 (336.6-375.7)	244.01 (226.89-261.62)	283.2 (263.1-303.3)	-0.83 (-0.87--0.8)
Socio-demographic index					
High	53.45 (51.2-55.05)	490.5 (470.4-505)	67.06 (61.79-70.71)	338.2 (316.8-354.9)	-1.28 (-1.32--1.25)
High-middle	44.16 (41.53-46.63)	436.8 (410-460.4)	70.8 (63.98-78.47)	364.6 (329.3-404.8)	-0.69 (-0.74--0.64)
Middle	31.43 (28.2-34.9)	273.6 (246.6-302.1)	71.21 (63.09-79.21)	259.4 (230.2-288.2)	-0.22 (-0.27--0.17)
Low-middle	10.21 (8.79-11.61)	149.4 (129-169.5)	25.64 (23.34-28.15)	166.5 (152-182.5)	0.37 (0.34-0.4)
Low	4.53 (3.51-5.21)	181.9 (142-208.3)	9.02 (8.00-10.14)	162.3 (145.3-181.9)	-0.49 (-0.59--0.39)
Sex					
Female	67.87 (62.93-73.04)	316.5 (292.9-340.4)	102.34 (92.58-110.65)	224.3 (203.2-242.7)	-1.25 (-1.3--1.19)
Male	76.1 (70.38-81.4)	405.6 (378.5-431.9)	141.67 (127.82-156.84)	349.7 (316.7-386.6)	-0.52 (-0.55--0.5)
Region					
High-income Asia Pacific	8.74 (8.37-9.05)	431.8 (411.8-447.5)	14.42 (12.72-15.5)	331.5 (303.8-352.5)	-0.93 (-0.98--0.88)
Central Asia	1.40 (1.32-1.49)	280.7 (263.9-298.7)	1.7 (1.52-1.88)	196.6 (175.6-216.7)	-0.88 (-0.99--0.76)
East Asia	36.92 (31.53-42.46)	389.6 (334.3-446.9)	71.49 (58.23-85.61)	334.5 (274-399.9)	-0.59 (-0.66--0.51)
South Asia	7.87 (6.76-8.89)	119.1 (101.9-135.2)	19.09 (17.12-21.55)	120.4 (108.3-135.3)	-0.1 (-0.2-0.01)
Southeast Asia	7.36 (6.12-8.42)	257.9 (218.1-292.2)	21.67 (18.69-24.56)	313.4 (270.7-353.5)	0.61 (0.55-0.67)
Australasia	1.34 (1.24-1.44)	577.9 (535.9-621.9)	1.67 (1.48-1.87)	327.2 (290.9-364.7)	-2.06 (-2.17--1.96)
Caribbean	0.84 (0.78-0.90)	317.3 (295.9-339.9)	1.8 (1.56-2.05)	335 (290.4-383.1)	0.33 (0.28-0.37)
Central Europe	7.67 (7.35-7.96)	512.4 (489.9-531.5)	10.88 (11.68-10.04-11.68)	506.5 (468-544.6)	-0.12 (-0.24-0)
Eastern Europe	12.81 (12.38-13.21)	455.7 (440.2-469.5)	14.65 (13.44-16.01)	424.5 (389.7-463.7)	-0.48 (-0.61--0.35)
Western Europe	28.15 (26.8-29.17)	498.6 (476.3-516.2)	29.12 (26.41-31.13)	326.8 (301.7-347.2)	-1.41 (-1.46--1.36)
Andean Latin America	0.44 (0.38-0.51)	203.5 (175.2-234)	1.36 (1.09-1.68)	226.8 (180.6-279.8)	0.39 (0.28-0.49)
Central Latin America	1.49 (1.44-1.54)	165.8 (159.2-171.3)	5.94 (5.29-6.62)	232 (206.6-258.6)	1.11 (1.03-1.18)
Southern Latin America	2.06 (1.9-2.24)	445.9 (410.2-484.8)	3.5 (3.11-3.92)	408 (363.1-457.6)	-0.04 (-0.19-0.1)
Tropical Latin America	2.18 (2.09-2.29)	224.3 (213.3-235.2)	7.45 (6.95-7.87)	286.2 (266.7-302.4)	0.77 (0.68-0.87)
North Africa and Middle East	4.1 (3.41-4.65)	221 (187.8-249)	10.13 (8.86-11.55)	209 (183.2-237.3)	-0.01 (-0.14-0.12)
High-income North America	16.13 (15.29-16.79)	469.4 (446.3-487.5)	19.08 (17.88-20.04)	316 (298.5-330.8)	-1.36 (-1.41--1.31)
Oceania	0.05 (0.04-0.06)	154.4 (127.4-186.6)	0.12 (0.1-0.14)	136.6 (116.1-159.4)	-0.39 (-0.48--0.3)
Central Sub-Saharan Africa	0.44 (0.35-0.55)	179.4 (145.6-220.2)	1.1 (0.83-1.47)	177.8 (135.2-238.6)	0.02 (-0.12-0.17)
Eastern Sub-Saharan Africa	2.25 (1.71-2.59)	274.5 (211.1-312.2)	4.44 (3.86-5.25)	242.1 (211.3-279.1)	-0.58 (-0.68--0.48)
Southern Sub-Saharan Africa	0.61 (0.55-0.72)	207.8 (185.4-251.1)	1.66 (1.5-1.87)	270.6 (244.9-301.9)	1.06 (0.79-1.34)
Western Sub-Saharan Africa	1.1 (0.94-1.31)	120.1 (102.6-141.3)	2.77 (2.25-3.28)	132.1 (110.4-155.5)	0.46 (0.4-0.52)

*ASR, age-standardised rate; EAPC, estimated annual percentage change; CI, confidence interval; UI, uncertainty interval.

Among five SDI regions, high SDI had the highest ASDR (15.0, 95% UI:13.6–15.9); high-middle SDI had the highest age-standardized DALYs (364.6, 95% UI:329.3–404.8). Only low-middle SDI saw rising ASDR (EAPC = 0.48, 95% CI:0.45–0.51) and DALYs (EAPC = 0.37, 95% CI:0.34–0.40). High SDI showed the steepest declines: ASDR EAPC=–1.31 (95% CI:–1.34 to –1.27); DALYs EAPC=–1.28 (95% CI:–1.32 to –1.25) (**Tables 2**, **3**).

**Figure 4:**
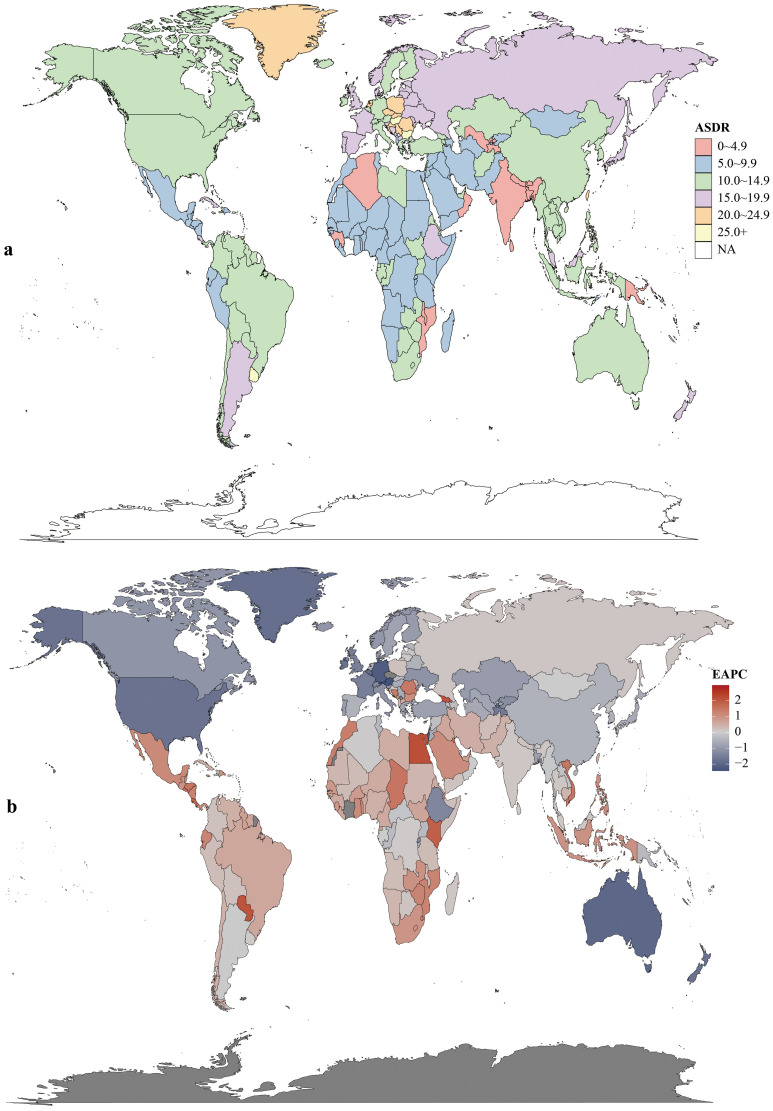
Mortality rates **(a)** and estimated annual percentage change (EAPC) **(b)** of colorectal cancer in 204 countries and territories.

Regionally, East Asia had the most CRC deaths throughout the period, totaling 287.88×10^3^ (95% UI:235.56×10^3^–343.28×10^3^) in 2021. Southern Sub-Saharan Africa saw the largest death increase (1990–2021; EAPC = 0.97, 95% CI:0.71–1.24). Highest ASDR were in Central Europe (22.6 per 100,000; 95% UI:20.8–24.3), followed by Eastern Europe and Southern Latin America (both 18.1 per 100,000). ASDR declined in 11 GBD regions (e.g., high-income Asia Pacific, East Asia, Western Europe). In 2021, the US, China, Japan had the most deaths; Tokelau, Niue, Nauru the fewest. Uruguay, Hungary, Bulgaria had highest ASDR; The Gambia, Papua New Guinea, Bangladesh the lowest (**Figure 4**, **Supplementary Table S1**).

**Figure 5:**
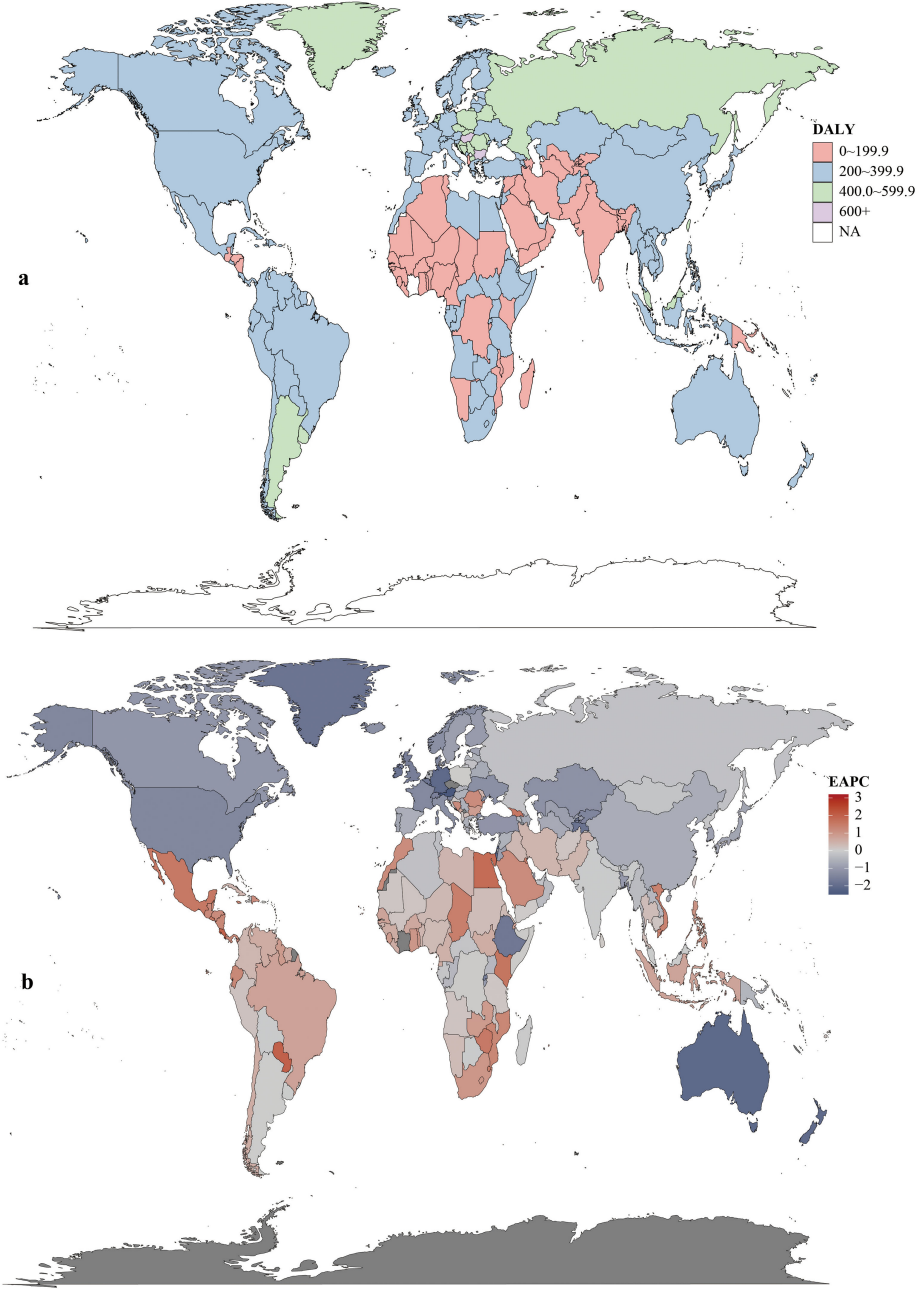
DALYs rates **(a)** and estimated annual percentage change (EAPC) **(b)** of colorectal cancer in 204 countries and territories.

Age-standardized DALYs rates rose in 15 regions and fell in 6. Highest rates were in Central Europe (506.5; 95% UI:468.0–544.6), Eastern Europe (424.5; 95% UI:389.7–463.7), and Southern Latin America (408.03; 95% UI:363.1–457.6). East Asia had the highest total DALYs (71.49×10^5^; 95% UI:58.23×10^5^–85.61×10^5^). Central Latin America saw the largest rate increase (1990–2021; EAPC = 1.11, 95% CI:1.03–1.18). In 2021, China had the highest total DALYs (6,848,389.9; 95% UI:5,513,406.6–8,284,228.3) and Hungary the highest rate (615.0; 95% UI:519.4–736.2). Lesotho had the largest DALYs increase (EAPC = 3.20, 95% CI:2.72–3.67); Maldives the largest decrease (EAPC=–2.49, 95% CI:–2.63 to –2.35) (**Figure 5**, **Supplementary Table S1**).

### Age and sex patterns

**Figure 6:**
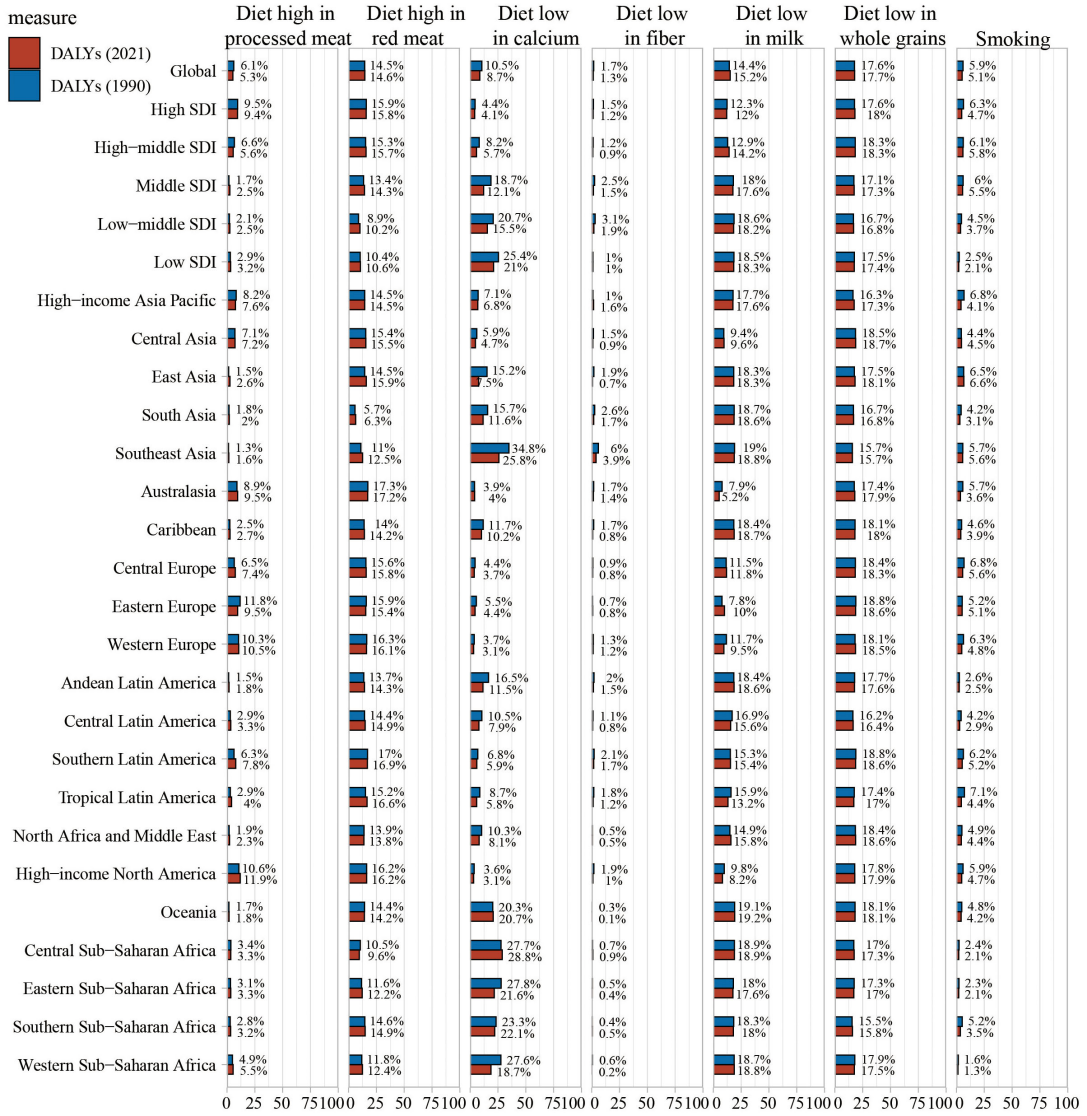
Changes in incidence rates in different age groups from 1990 to 2021.

In 2021, global CRC incidence peaked in the 90–94 age group, rising consistently with age. Versus 1990, 2021 rates increased in 25–74 and 85+ groups but stabilized in 75–84 (**Figure 6**).Across most SDI regions, incidence rose in all age groups, except high SDI where 55–89 age rates declined. High SDI had higher rates overall, particularly ≥40 years, with rapid increases after 40 (**Figure 1**). Incidence was higher in women globally, but men in high, high-middle, and middle SDI regions showed marked acceleration (**Figure 1**). Mortality rates peaked in the ≥ 95 age group (**Supplementary Figure S1**), and DALY rates followed the same age pattern (**Supplementary Figure S2**). Global 2021 mortality rates were lower than in 1990 and rose with age. High SDI regions saw reduced mortality and DALY rates over 32 years; other SDI regions had increases.

Notably, early-onset CRC (EO-CRC, defined as CRC in adults aged 25–49 years) showed a significant upward trend. By 2021, incidence in the 25–74 age group (which includes EO-CRC) had increased notably compared to 1990, with clear regional differences by SDI. In high SDI regions, EO-CRC in 25–49-year-olds continued to rise despite falling incidence in 55–89-year-olds. In middle-low SDI regions, EO-CRC growth was more prominent—even outpacing late-onset CRC (LO-CRC, in adults ≥ 50 years) in some areas. Young men (35–49 years) also faced a higher EO-CRC risk than women across multiple SDI regions.

### Risk factors

**Figure 7:**
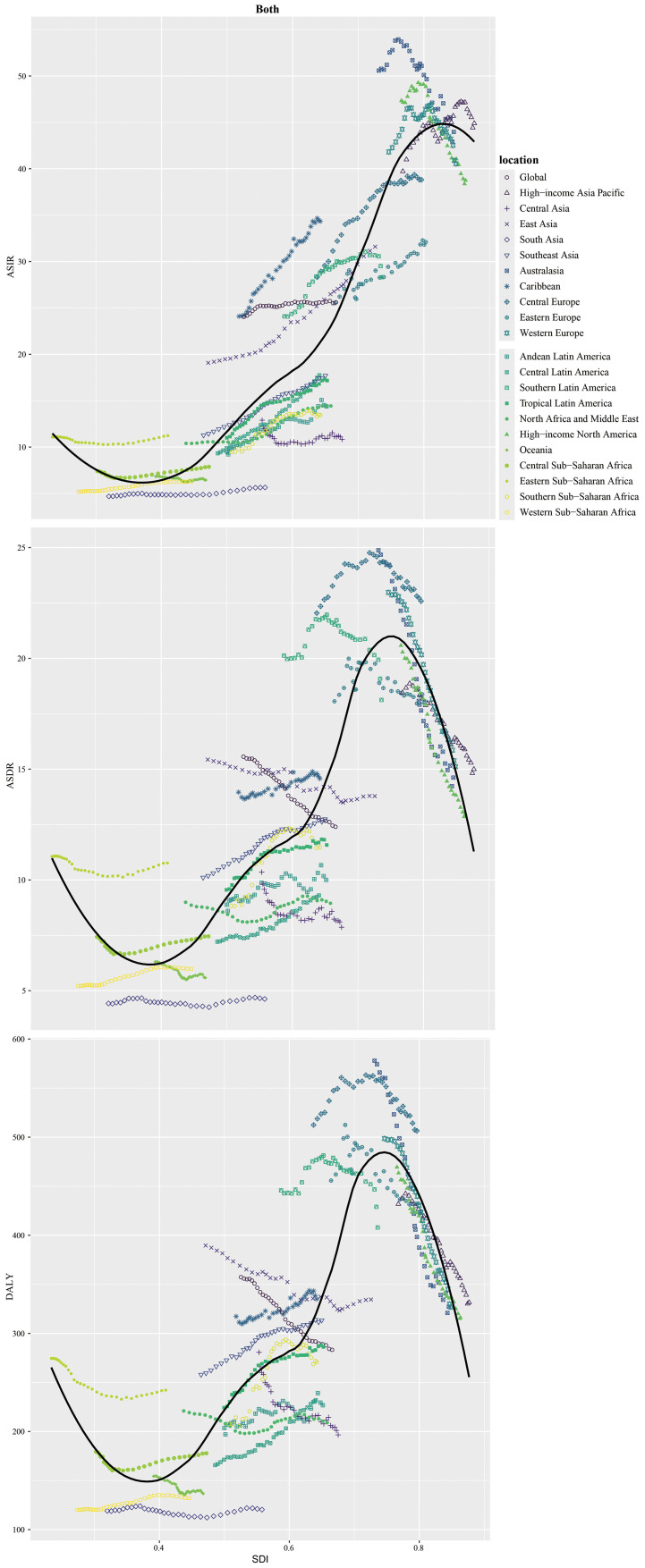
Percentage contribution of risk factors to all-age DALYs of colorectal cancer in 2021, for both sexes, globally and by regions.

According to the risk factor classification in the GBD database, CRC risk factors are categorized into three levels (**Figure 7**, **Supplementary Figure S3**, **Supplementary Figure S4**). Globally, dietary risks accounted for the most DALYs, followed by high BMI, high fasting plasma glucose, and alcohol use. Regional primary factors differed: milk-poor diets led in high-income Asia Pacific, East Asia, South Asia, the Caribbean, Andean Latin America, and Western Sub-Saharan Africa; low whole-grain diets in Central Asia, Australasia, European regions, most Latin American regions, North Africa and the Middle East, and high-income North America; and calcium-poor diets in Southeast Asia, Oceania, and other Sub-Saharan African regions.

In 2021, tobacco exposure was responsible for 5.1% of CRC-related disability-adjusted life years (DALYs), representing a decline from preceding years. Within high SDI regions, the proportion of CRC DALYs attributable to tobacco stood at 4.7%. Across the remaining 21 Global Burden of Disease (GBD) regions, the share of CRC DALYs linked to smoking also decreased in comparison to 1990 figures. In contrast, a diet deficient in whole grains accounted for 17.7% of CRC DALYs in 2021, exhibiting a slight upward trend relative to previous years. Among the high SDI quintile, elevated fasting plasma glucose contributed to 18.0% of CRC DALYs, with a similarly modest increasing trajectory observed. (**Supplementary Figure S4**).

Globally, the contribution of a whole-grain-poor diet to CRC DALYs varied across SDI quintiles in 2021. In high and high-middle SDI regions, a diet low in whole grains was the leading contributor (17.7% in high SDI; 18.0% in high-middle SDI). In middle and low-middle SDI regions, a milk-poor diet was the primary contributor (17.6% in middle SDI; 18.2% in low-middle SDI), whereas a calcium-poor diet was the top contributor in the low SDI region (21.0%). Overall, CRC deaths attributable to behavioral risks decreased compared with 1990, while those linked to metabolic risks increased (**Supplementary Figure S3**).

## Discussion

This study provides a comprehensive analysis of the global Colorectal cancer (CRC) burden from 1990 to 2021, highlighting significant disparities across SDI (Socio-demographic Index) regions. The global CRC incidence more than doubled over 32 years, consistent with previous research on rising global CRC trends (17, 18).

Between 1990 and 2021, incidence and mortality rates declined in high SDI countries but rose in some low-middle and middle SDI regions. Low, low-middle, middle, high-middle, and high SDI regions saw a 291.4% increase in incident cases (from 76.7% of the global total) and a 169.3% increase in deaths (from 38.4% of global deaths). The proportion of global DALYs (disability-adjusted life years) from these regions grew from 25.5% to 151.1%.

We observed substantial increases in ASIR (age-standardized incidence rates) between 1990 and 2021 across Asia—for instance, 65.4% in East Asia and 56.6% in Southeast Asia—and Latin America, including 90.3% in Central Latin America and 56.4% in Tropical Latin America.

Driven by rapid economic growth and industrialization, the expanding middle class in developing nations has adopted Westernized lifestyles marked by unhealthy dietary patterns (low fruit and vegetable intake, high consumption of red and processed meats), sedentary behaviors (e.g., prolonged television viewing), reduced physical activity, and substance use (alcohol and tobacco) (19). Such behavioral shifts contribute to the rising incidence of lifestyle-related diseases, including CRC.

Furthermore, advancements in medical screening technologies have also contributed to higher detection rates of CRC in high-middle SDI regions (20–24). Conversely, owing to constrained healthcare resources and limited medical expertise, many CRC cases in low SDI regions remain undiagnosed or unrecorded (17, 25). While the current burden of CRC in lower SDI regions is relatively low, attention must be paid to rising trends in alcohol consumption and metabolic risk factors. Recent research highlights significant increases in metabolic disorders and alcohol-related complications in these regions (26–28), which may drive a future surge in CRC cases. Thus, overly optimistic conclusions about the current burden are unwarranted. Proactive public health interventions and sustained monitoring are critical to mitigating these emerging risks in vulnerable populations (18).

The CRC screening guidelines adopted by different countries may influence the disease burden of CRC. Most nations recommend initiating CRC screening for the general risk population at age 50 (21, 29–31). With growing attention to CRC, China’s CRC Screening Guidelines lowered the screening initiation age for the general risk population to 40 in 2020 (32). Similarly, the US Preventive Services Task Force (USPSTF) revised its recommendation in 2021, reducing the initial CRC screening age from 50 to 45 (33, 34). Japan, by contrast, has mandated routine CRC screening for adults aged 40 and above since as early as 1992 (29). Our findings reveal that the mortality rates of CRC in Japan, China, and the United States rank significantly lower than their respective incidence rates. Studies indicate that between 1990 and 2019, Japan exhibited the lowest CRC mortality-to-incidence ratio across Asia (35). This suggests that while early screening may not reduce the incidence of CRC, it could mitigate the risk of fatal outcomes associated with the disease. However, these findings require further research to be confirmed.

Our study revealed that while the incidence, mortality, and DALYs related to CRC have risen sharply over the past three decades, their proportions relative to the overall burden of CRC have been on the decline. This outcome indicates that the growth rate of the disease burden associated with CRC is relatively slower compared to that of all CRC cases. Such findings are consistent with those reported in other studies (20, 36). A potential explanation lies in the fact that age is a well-established risk factor for CRC, and this is compounded by the effects of increased life expectancy and a growing aging population (37). As a result, CRC in elderly populations has emerged as a critical public health issue demanding targeted attention.

Data from the study shows that early-onset CRC (EO-CRC, 25–49 age group) incidence rose significantly, with 25–74 age group up notably by 2021 vs 1990 and SDI-based regional differences. High-SDI regions saw EO-CRC rise in 25-49s despite lower 55-89s incidence; middle-low SDI regions had more obvious EO-CRC growth, and young men were at higher risk. Young people’s behavioral risks may drive this. With insufficient research on EO-CRC mechanisms in youth, targeted interventions and lower screening age are needed to reduce its burden.

The precise cause behind the rising incidence of CRC in individuals under 50 remains elusive, but one plausible explanation may lie in the birth cohort effect: those born in the latter half of the 20th century have faced growing exposure to modifiable behavioral risk factors, including unhealthy dietary patterns, obesity, sedentary lifestyles, reduced physical activity, and elevated smoking rates during early adulthood (14). However, most of these risk factors have been identified based on evidence derived from CRC patients aged 50 or older, leaving the exact mechanisms and underlying risk factors for younger populations relatively unclear (38–41).

Notable disparities exist in the disease burden of CRC across continents and countries, even among regions with similar SDI levels. These differences may arise from variations in population size, lifestyle trends, ethnic genetics, and healthcare access. Prior research highlights the significant role of ethnicity in CRC development within East Asia: certain ethnic groups, such as the Chinese, Koreans, and Japanese, exhibit higher incidence rates. Even in ethnically diverse countries like Singapore and Malaysia—where different racial groups share similar environmental conditions and dietary habits—Chinese populations show a significantly elevated risk of CRC compared to Malays and Indians. Additionally, within the same ethnic group in China, individuals residing in coastal areas experience higher incidence and mortality rates than their inland counterparts (42, 43).

A major limitation of this study is the lack of cancer registry data in many African, Caribbean, and Asian countries. While GBD uses available data from vital registries, oral autopsy, etc., the accuracy of its estimates is heavily dependent on cancer registry data, which is lacking or inadequately covered in many low - and middle-income countries in sub-Saharan Africa and Asia, with underreporting due to poor documentation, inadequate facilities/staff, etc. In addition, 95% ui (uncertainty interval) cannot account for bias such as measurement or selection bias. Second, because of the longer statistical period, inadequate disease registration up to 30 years ago (especially in low - and middle-income countries) may have biased earlier (1990s) estimates, as reflected in the wide 95% ui. Third, delays in reporting cancer data in 2021, even in regions with high SDI, led to recent trend-based estimates using a broader ui.

In conclusion, global CRC incidence and mortality have more than doubled in three decades. Mitigating modifiable risks and boosting screening are key to reducing deaths. Low and middle SDI regions (accounting for ~75% of DALYs) will see further rises due to aging, longer lifespans, better detection, and lifestyle shifts. Strategies like lifestyle adjustments, early screening, quality healthcare, and advanced treatments are critical. Population-based registries aid monitoring. The rising incidence in those <50 is a warning, requiring greater awareness and research into its drivers.

Data generated by the Global Burden of Disease study serves as a vital resource for both the general public and healthcare providers, as it illuminates the effectiveness of current treatment strategies, the impacts of past interventions, and the necessity of implementing preventive measures. The findings from GBD 2021 not only offer actionable insights for policymakers but also provide fresh perspectives for scientists and physicians worldwide. These comprehensive and comparable estimates can inform efforts to advance equitable CRC control on a global scale, with the overarching goal of alleviating the global cancer burden.

## Data Availability

Publicly available datasets were analyzed in this study. This data can be found here: https://ghdx.healthdata.org/gbd-2021.

## Supplementary material

Supplementary Figure 1 Changes in mortality rates in different age groups from 1990 to 2021.

Supplementary Figure 2 Changes in DALYs rates in different age groups from 1990 to 2021.

Supplementary Figure 3 Level-1: Percentage contribution of risk factors to all-age DALYs of colorectal cancer in 2021, for both sexes, globally and by regions.

Supplementary Figure 4 Level-2: Percentage contribution of risk factors to all-age DALYs of colorectal cancer in 2021, for both sexes, globally and by regions.

Supplementary Figure 5 Age-standardized rates (per 100,000 population) in female of colorectal cancer among regions based on SDI in 2021. **(a)** ASIR. **(b)** ASDR. **(c)** Age-standardized DALY rate.

Supplementary Figure 6 Age-standardized rates (per 100,000 population) in male of colorectal cancer among regions based on SDI in 2021.**(a)** ASIR. **(b)** ASDR. **(c)** Age-standardized DALY rate.
