# Supplementary material for: The global, regional, and national burden of colorectal cancer and its attributable risk factors in 204 countries and territories, 1990-2021: a systematic analysis for the global burden of disease study 2021
Source: Front Oncol. 2025 Nov 19;15:1665430. doi: 10.3389/fonc.2025.1665430 (PMC12672353; doi:10.3389/fonc.2025.1665430)
Supplement: Supplementary Figure 1 — Changes in mortality rates in different age groups from 1990 to 2021. [file DataSheet1.pdf]

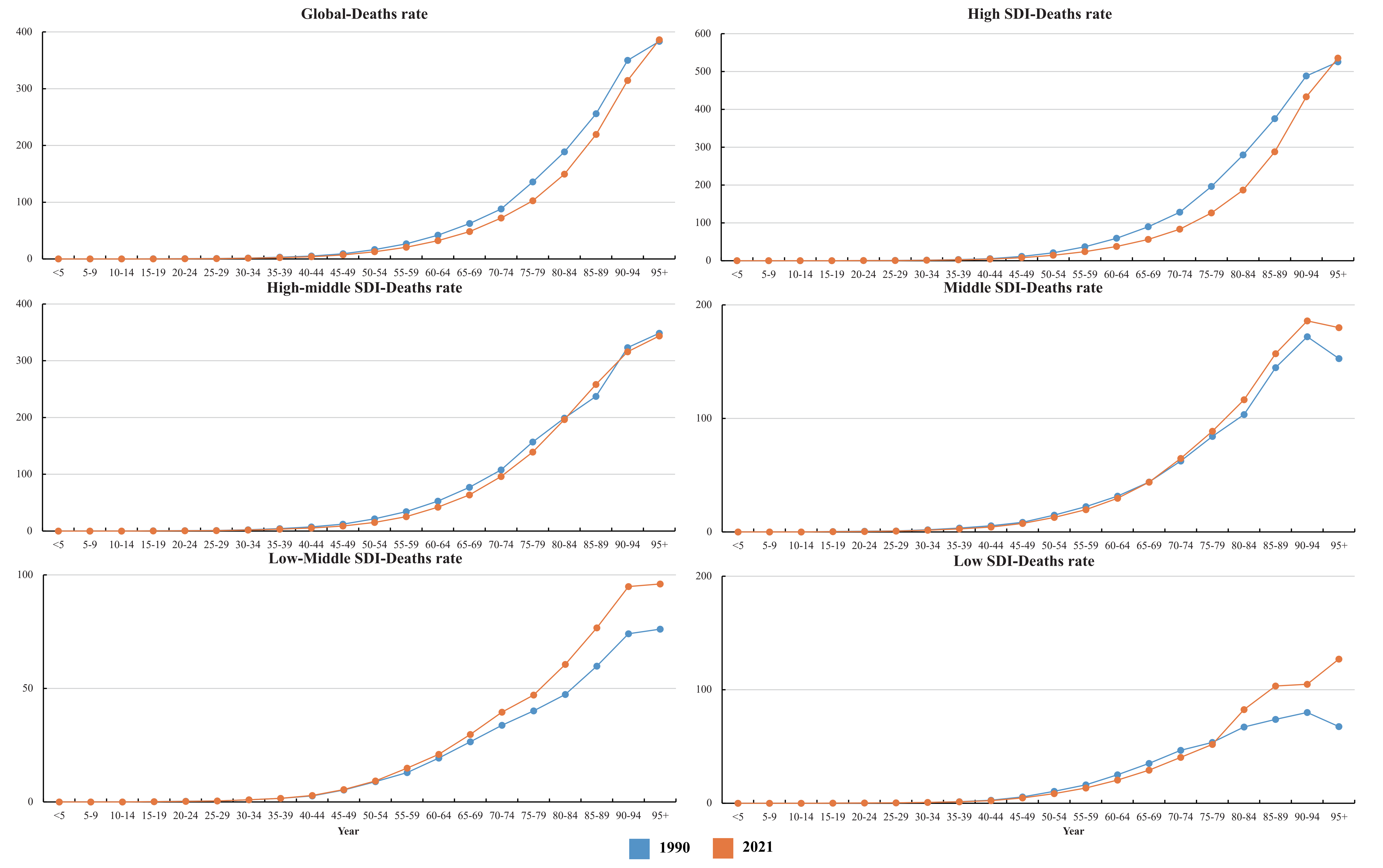

Global-Deaths rate
High SDI-Deaths rate
High-middle SDI-Deaths rate
Middle SDI-Deaths rate
Low-Middle SDI-Deaths rate
Low SDI-Deaths rate
<5
5-9
10-14
15-19
20-24
25-29
30-34
35-39
40-44
45-49
50-54
55-59
60-64
65-69
70-74
75-79
80-84
85-89
90-94
95+
Year
1990
2021
