# Supplementary figures and images for: The global, regional, and national burden of colorectal cancer and its attributable risk factors in 204 countries and territories, 1990-2021: a systematic analysis for the global burden of disease study 2021
Source: Front Oncol. 2025 Nov 19;15:1665430. doi: 10.3389/fonc.2025.1665430 (PMC12672353; doi:10.3389/fonc.2025.1665430)

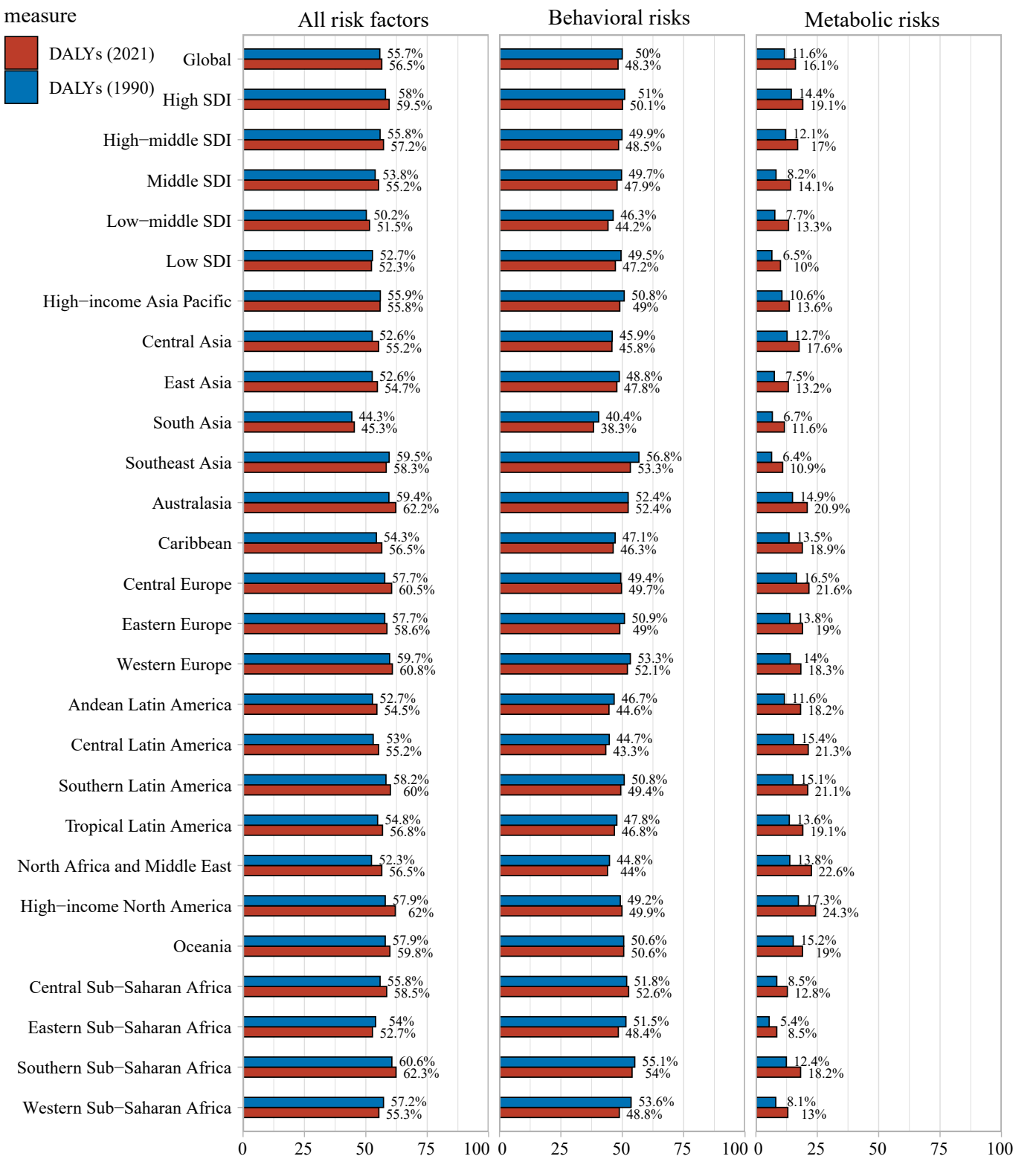

Supplement: Supplementary Figure 3 — Level-1: Percentage contribution of risk factors to all-age DALYs of colorectal cancer in 2021, for both sexes, globally and by regions. [file DataSheet3.pdf]

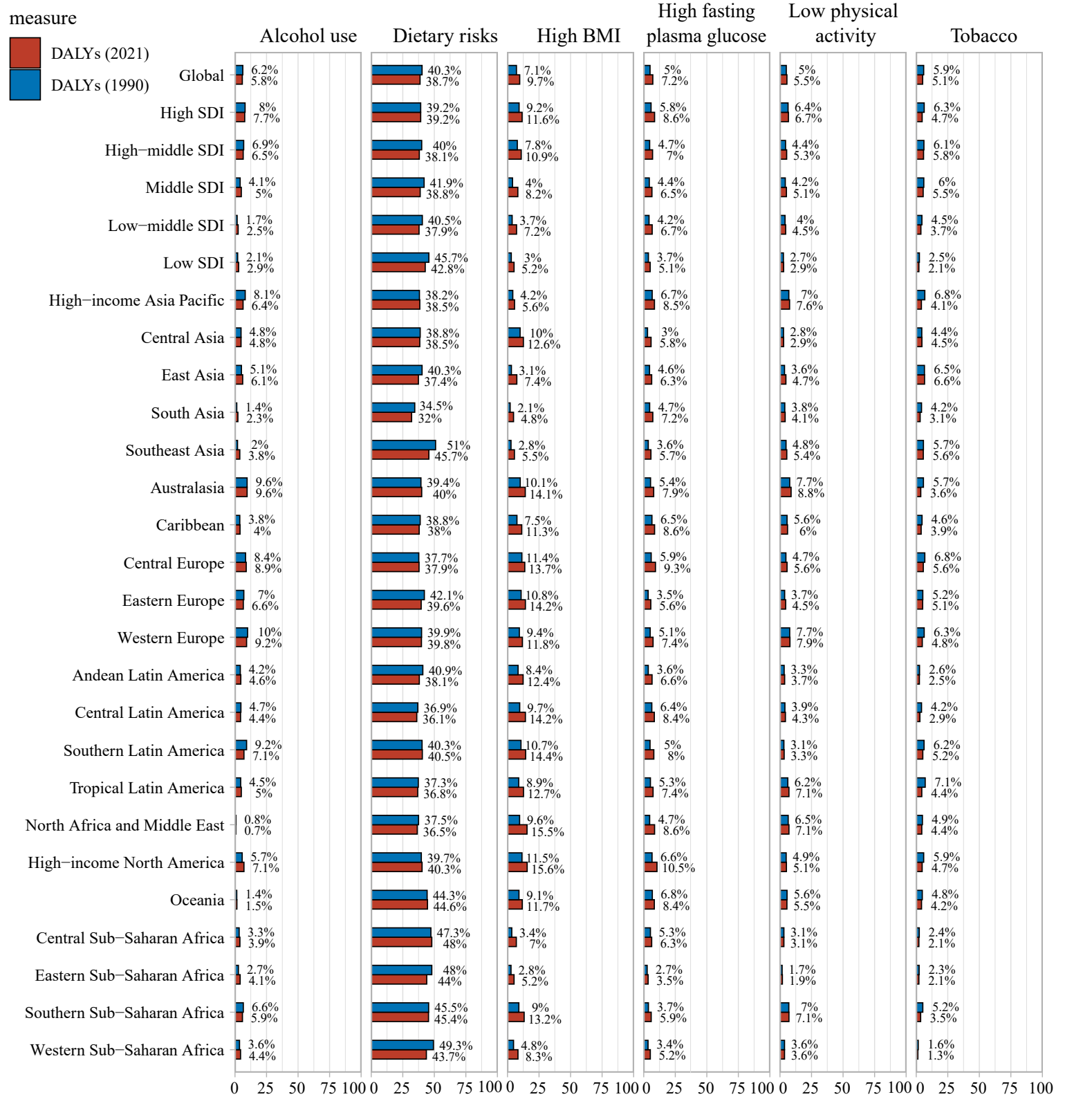

Supplement: Supplementary Figure 4 — Level-2: Percentage contribution of risk factors to all-age DALYs of colorectal cancer in 2021, for both sexes, globally and by regions. [file DataSheet4.pdf]

Female

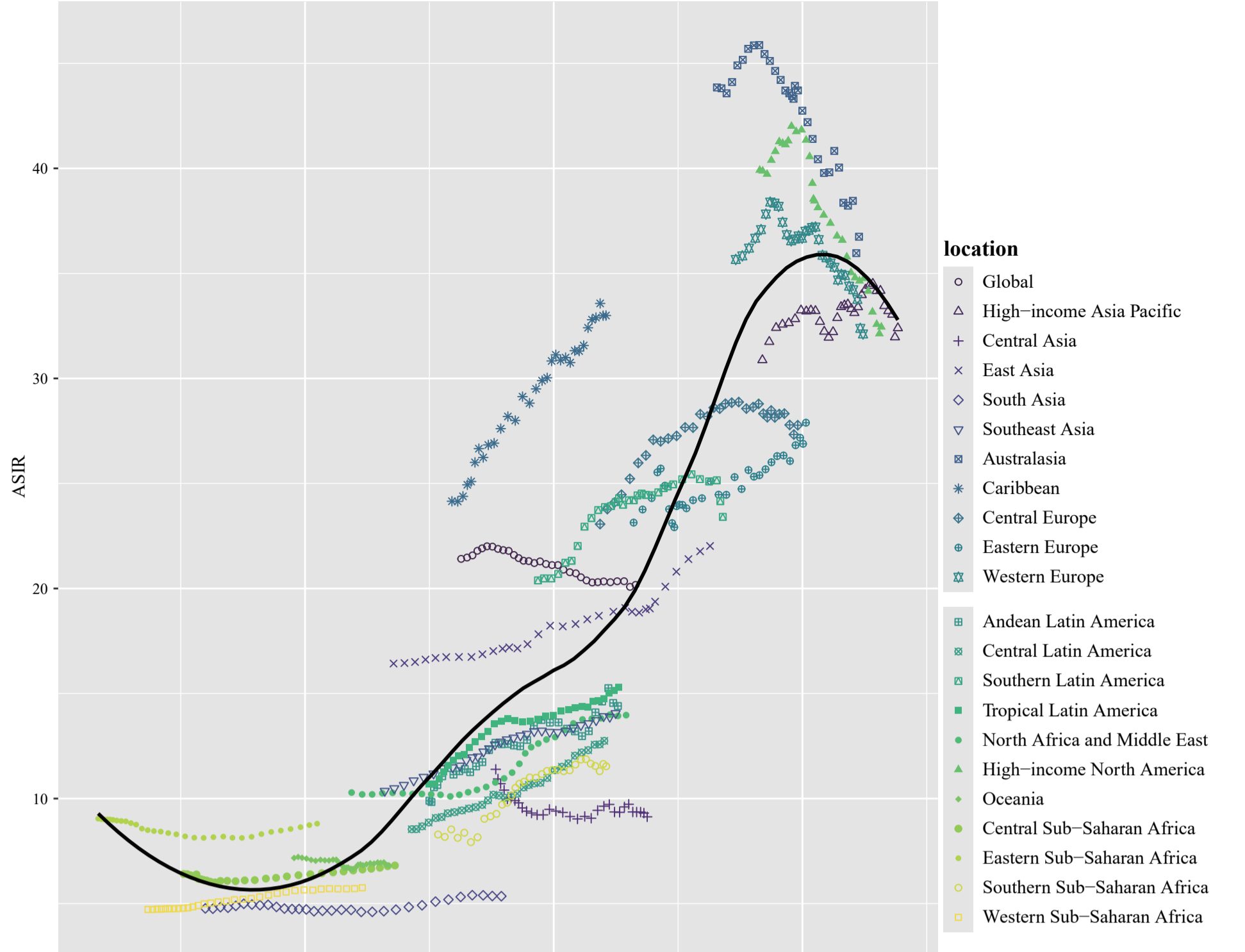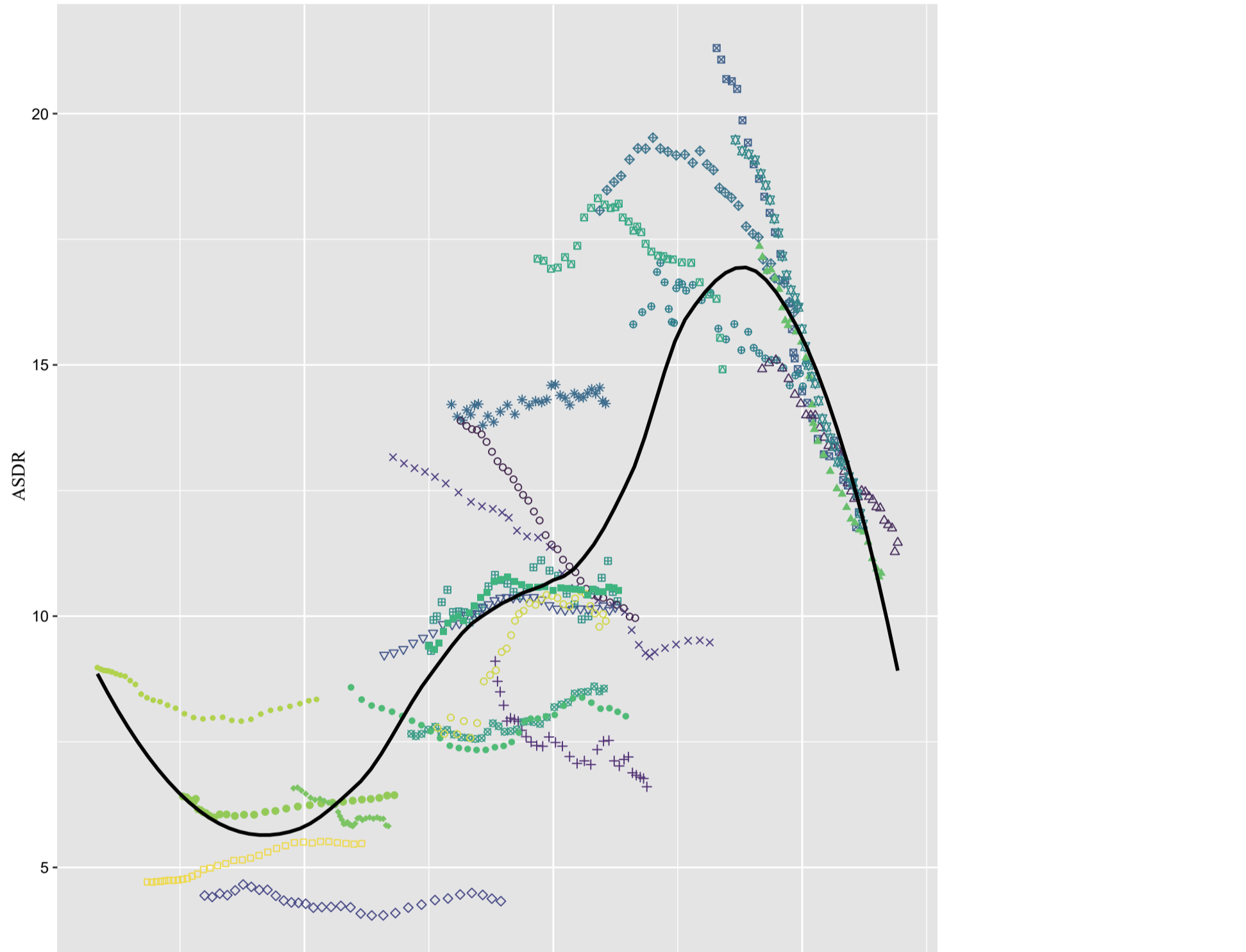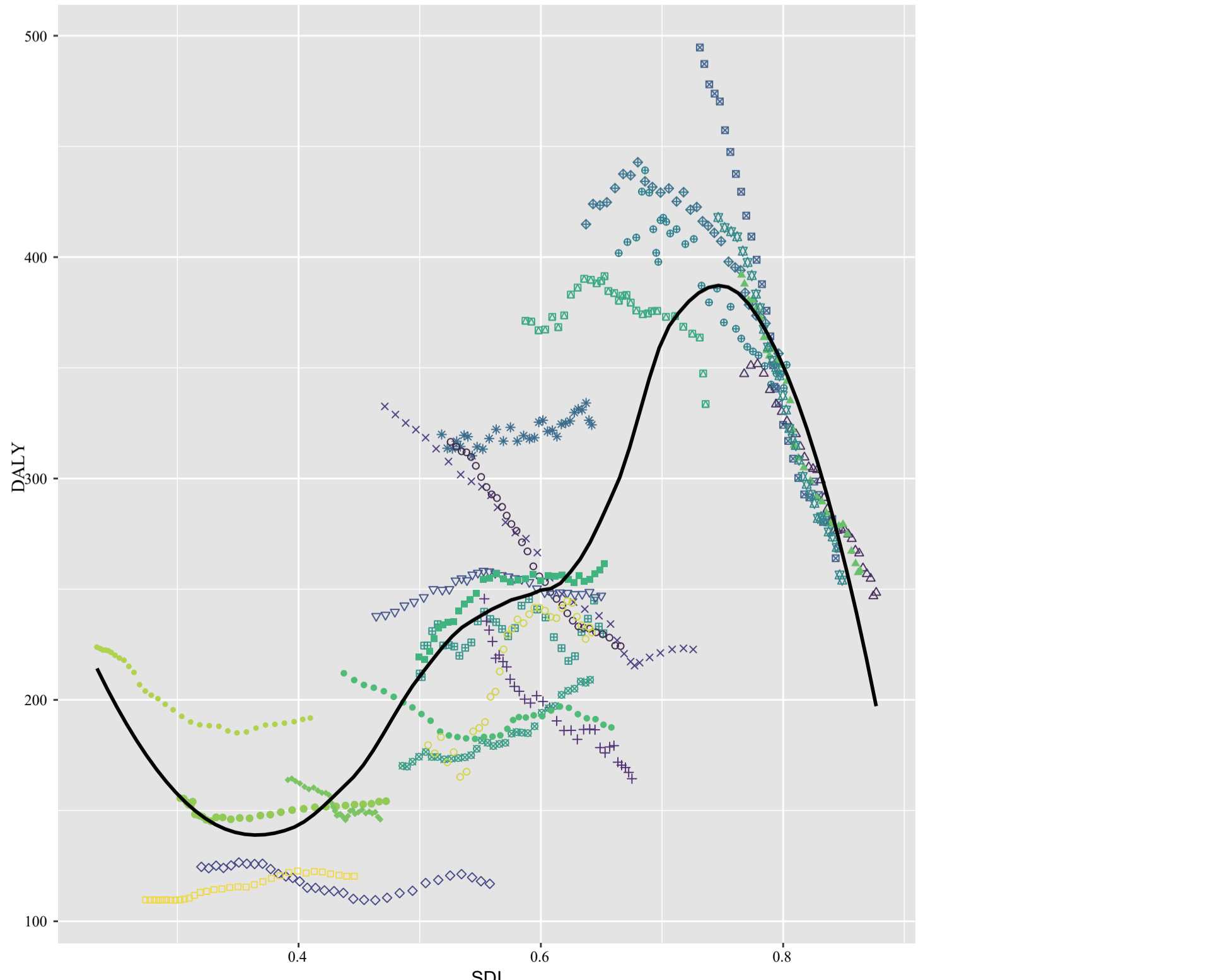

Supplement: Supplementary Figure 5 — Age-standardized rates (per 100,000 population) in female of colorectal cancer among regions based on SDI in 2021. (a) ASIR. (b) ASDR. (c) Age-standardized DALY rate. [file DataSheet5.pdf]

Male

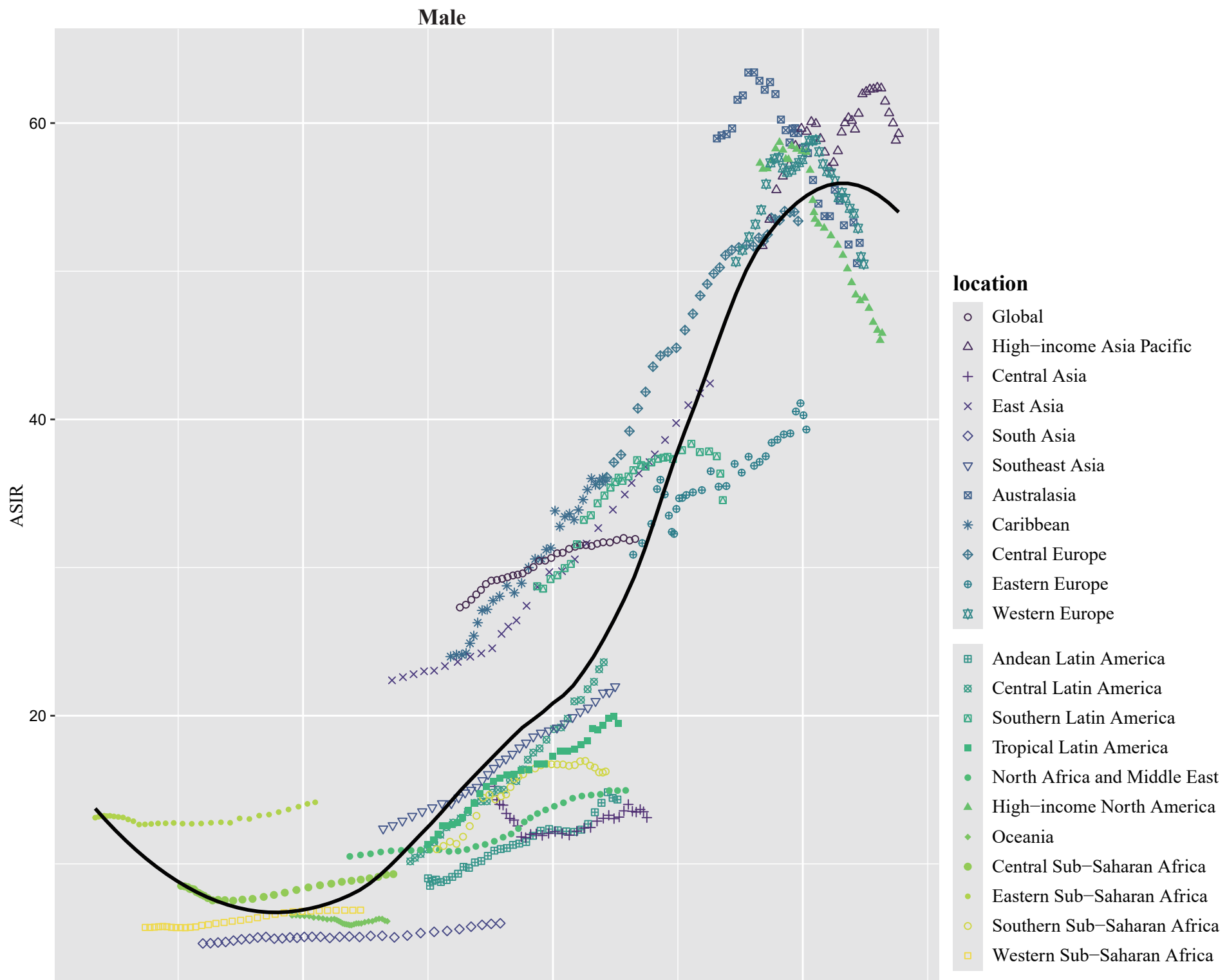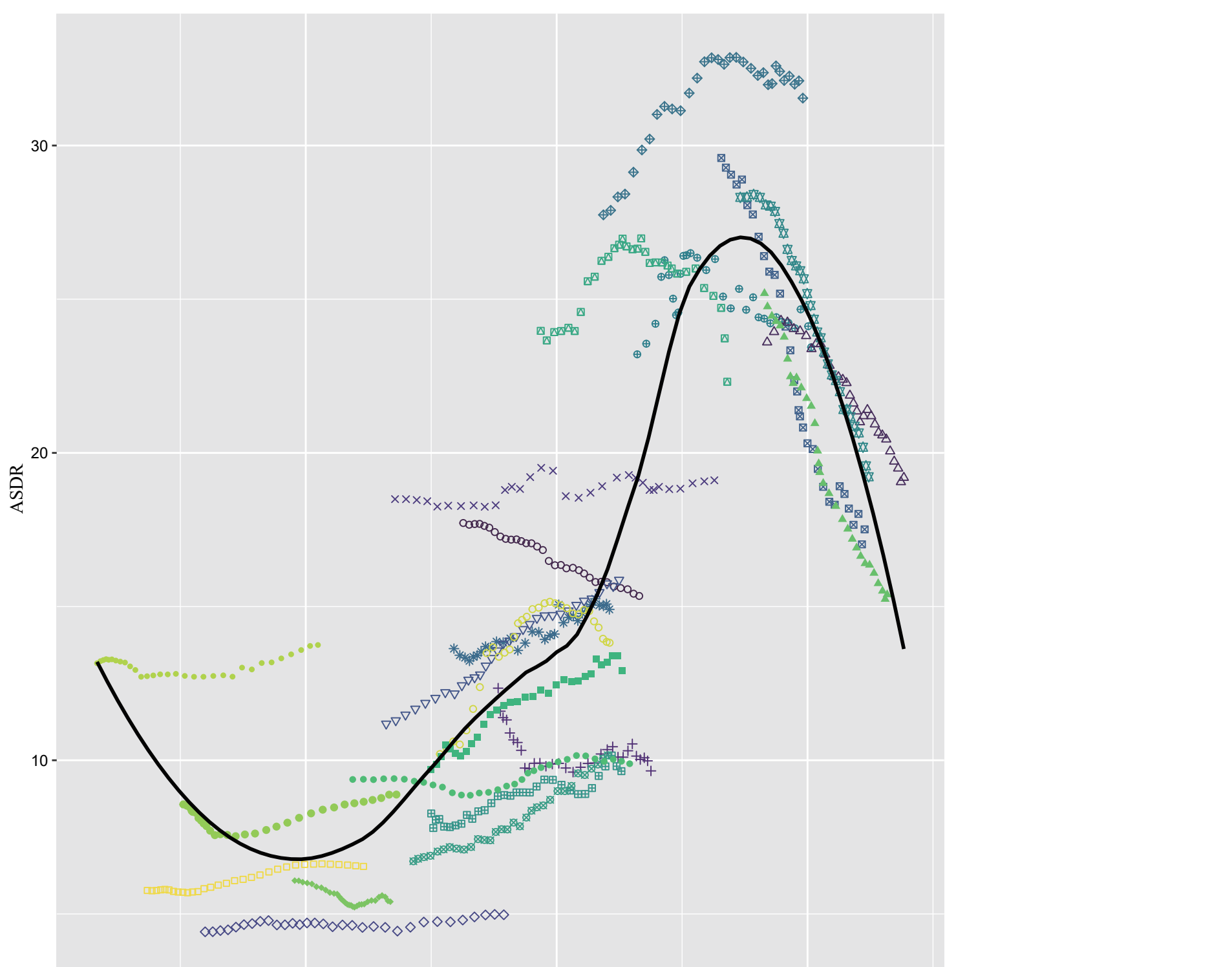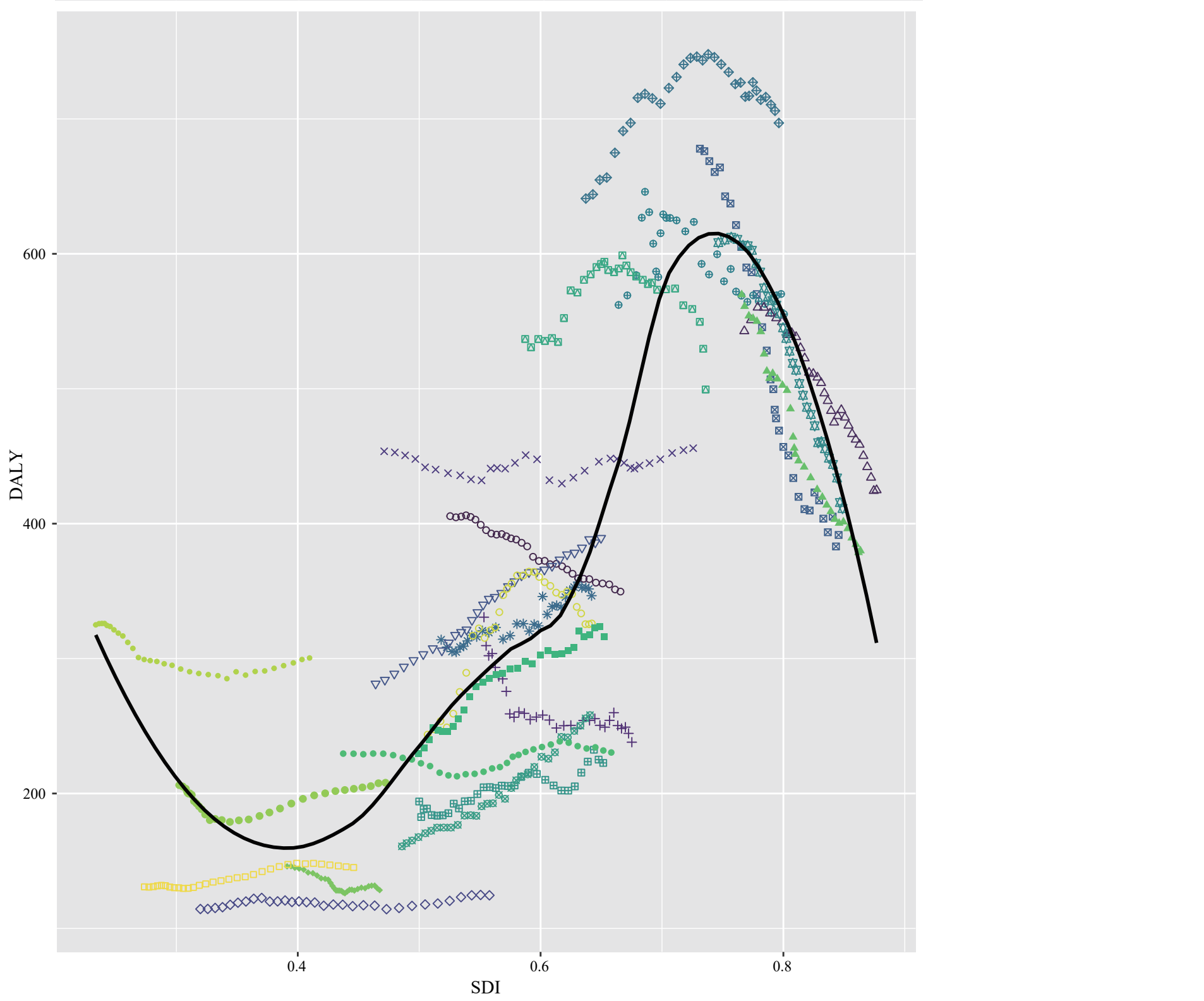

Supplement: Supplementary Figure 6 — Age-standardized rates (per 100,000 population) in male of colorectal cancer among regions based on SDI in 2021.(a) ASIR. (b) ASDR. (c) Age-standardized DALY rate. [file DataSheet6.pdf]
